# AI-Driven Lumped-Element Modeling of Human Respiratory System for Studying Voice Mechanics

**DOI:** 10.21203/rs.3.rs-9119279/v1

**Published:** 2026-06-25

**Authors:** Maruf Md Ikram, Maryam Naghibolhosseini, Mohsen Zayernouri

**Affiliations:** 1 Department of Mechanical Engineering, Michigan State University, East Lansing, MI 48824, USA; 2 Department of Communicative Sciences and Disorders, Michigan State University, East Lansing, MI 48824, USA

**Keywords:** Multi-Physics Modeling, Data-Driven Dynamics, Voice Biomechanics, High-Speed Videoendoscopy

## Abstract

A predictive physics-based model of human respiratory, phonatory, and articulatory subsystems is developed to simulate voice production. Representing lungs, compressible airways, and vocal folds as spring-damper-mass controlled piston-cylinder systems, our mathematical model robustly captures the intricate dynamics of airways during phonation. The nonlinear viscoelastic properties of lung tissues and compressible airways were investigated, yielding a responsive and expressive baseline respiratory model with the capability to further extend into a patient-specific model for both respiration and phonation. The resulting framework was subsequently integrated with a mechanical representation of the vocal tract, governed by the glottal area waveform (GAW) capturing the motion of vocal folds during sustained phonation. The GAW is extracted from laryngeal high-speed videoendoscopy data of a normophonic participant using deep learning. Our novel paradigm transcends beyond modeling the respiratory system, enabling AI-driven modeling of vocalization, including vocal fold dynamics, interactions with flow aerodynamics, and flow resistances, induced by the oscillatory behavior of vocal folds. Our investigation leads to the first-ever simulation of respiratory dynamics for vocalization, directly advancing the prediction of subglottal pressure distributions, impossible to measure directly and noninvasively in humans, dynamic resistances, and energy transfer mechanisms during phonation in voice mechanics.

## Introduction

Human voice production is a complex process emerging from the closely coupled interactions of the respiratory, phonatory, and articulatory subsystems. The respiratory subsystem generates the driving force for phonation by regulating airflow and subglottal pressure through the biomechanics of lung expansion/contraction, and airways flow resistances^1^. The phonatory subsystem, within the larynx, interacts with the airflow, leading to self-sustained oscillations of vocal folds and producing a quasi-periodic acoustic signal^2^. Finally, the articulatory subsystem, consisting of the supraglottal vocal tract influences the voice by creating resonances and anti-resonances via changing its shape^3^. Each subsystem contributes distinctively to phonation by their interdependent but collaborative dynamics. Hence, to create an accurate model of phonation, these subsystems should collectively be included within the modeling framework. Despite this interdependence, computational models of phonation have historically only emphasized the phonatory and articulatory subsystems. As a result, these models often considered a uniform subglottal inlet condition using an averaged or sinusoidal flow to drive the pressure profiles^4^. Although such simplifications capture gross features of glottal flow, they overlook subject-specific respiratory mechanics and fail to reproduce the irregularities, asymmetries, and nonlinear variations inherent in true phonation dynamics. Physiological and experimental studies have shown that the glottal and supraglottal flow fields are highly sensitive to inlet conditions, and even small variations in subglottal pressure/velocity can alter jet asymmetry, turbulence intensity, and acoustic loading^1,5,6^. To address these challenges, the present study focuses on developing a holistic lumped-element model, including all voice production subsystems, to generate subject-specific inlet conditions, essential for developing accurate and clinically meaningful computational models of phonation.

As the airflow during exhalation enters the glottis, it develops into complex flow phenomena including jet formation, vortex shedding, turbulence, and the Coandă effect, all of which shape the resulting sound field and phonation stability^7,8^. These behaviors can be visualized most clearly in computational modeling studies, which provide access to detailed velocity fields, pressure distributions, and temporal flow evolution that cannot be captured in vivo in humans due to experimental limitations^9–11^. Such computational models have revealed that glottal jet asymmetry, supraglottal recirculation, and acoustic loading are strongly dependent on how the inlet condition, especially it’s temporal evolution, is prescribed^12–15^. Therefore, including a realistic inlet condition is critical toward having a robust and stable model of phonation. This underscores the need for considering the respiratory subsystem modeling to generate subject-specific time-dependent boundary conditions for the larynx. While researchers have recently incorporated time-dependent inlet conditions in phonation modeling, the implementation was done using a uniform subglottal pressure/velocity^16–19^. These studies showed the glottal jet could still develop asymmetries, producing lateral skewing, wall attachment consistent with the Coandă effect, and vortex structures altering acoustic loading and phonatory stability^12,20,21^. Later investigations demonstrated that even slight time-dependent non-uniformities at the inlet could significantly change the jet trajectory, turbulence onset, and the strength of supraglottal vortical structures^13,20,22^. More recently, the (semi-)sinusoidal inlet conditions presenting a time-varying pressure/velocity at the subglottal boundary were introduced to approximate the impact of rhythmic breathing in voice production^23,24^. While this represented a progress over the use of a steady inflow, such sinusoidal inputs imposed artificial regularity and could not reproduce the nonlinear and irregular characteristics of true subglottal dynamics^25^. Moreover, sinusoidal conditions are idealistic and therefore disregard subject-specific variations linked to tbe respiratory effort, vocal fold vibratory dynamics, and supraglottal flow resistance.^26–28^. These limitations highlight the need for more advanced approaches that can generate inlet conditions based on the subject’s respiratory function rather than relying on idealized/averaged assumptions.

Lumped-element models (LEMs) have been used to study respiratory mechanics during breathing^29–32^. Early work by Sharp et al. established methods to quantify respiratory compliance and resistance from pressure-volume relationships^32^. Later, Athanasiades et al. developed a nonlinear LEM of the respiratory system^31^. Later, Bersani et al. proposed a Windkessel-type multi-compartment 0-D models of lung and airways mechanics dividing the respiratory system into the rigid upper airways, the compliant intrapleural space, and the alveolar gas-exchange region^30^. Their framework explicitly coupled airways flow resistance, tissue compliance, and alveolar gas dynamics, highlighting the suitability of lumped models for patient-specific applications. More recently, Marconi and Lazzari expanded the model of respiration by Athanasiades et al. and applied it to healthy and pathological conditions, capturing essential features of breathing cycles, such as alveolar volume changes, intrapleural pressure dynamics, and flow-volume loops^29^. These studies established LEMs as a justifiable approach to capture pressure-flow-volume behaviors of the respiratory system.

While LEMs have been used to model lung/airways mechanics, their applications have been limited to breathing. In this study, we present the first LEM that extends beyond modeling respiration and explicitly captures phonation mechanics of human’s sustained phonation production. Our approach models the function of the lungs and respiratory system, with active dynamic vocal folds, bridging the gap between pulmonary airflow models and phonatory dynamics. This approach leads to an accurate and robust reconstruction of time-dependent glottal inlet conditions, generating a subject-specific computational model of vocalization. This work establishes a benchmark study with subject-specific data from a normophonic participant, which creates a foundational model that can be adjusted in the future to generate the subglottal conditions for various pathologies affecting voice production.

## Results

**Figure 1.**
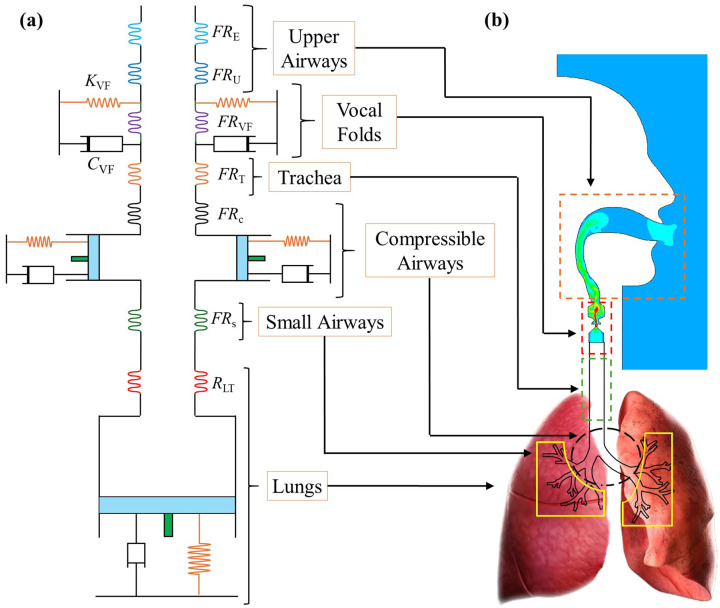
Unified mechanical-physiological representation of the human phonation system. **(a)** Lumped-element modeling framework with three coupled subsystems: respiratory (lungs and compressible airways), phonatory (vocal folds), and articulatory (upper airways). Airflow generated by lung contraction propagates through the small airways, compressible airways, vocal folds, and upper airways before exiting at the mouth, encountering lung tissue resistance $R_{\text{LT}}$ and several flow resistances $FR_{\text{s}},FR_{\text{c}},FR_{\text{T}},FR_{\text{VF}},FR_{\text{U}}$, and $FR_{\text{E}}$. **(b)** Anatomical correspondence of the respiratory, phonatory, and articulatory subsystems, mapping the lumped-element model to human airway anatomy.

In this work, we develop a unified lumped-element model of human phonation that integrates the respiratory, phonatory, and articulatory subsystems within a single mechanical framework, as illustrated in Fig. 1. The model represents the airflow pathway from the lungs to the atmosphere using spring-mass-damper elements along with distributed flow resistances. The flow resistances quantify the pressure loss per unit volumetric flow rate and account for energy dissipation across different anatomical segments. The respiratory subsystem comprises both the lungs and the compressible airways, with the two lungs combined into a single equivalent cylindrical chamber. Volumetric changes of this chamber are modelled by piston motion governed by a spring-mass-damper system. The airflow generated by the lungs first encounters the resistance due to lung tissue deformation $\left(R_{\text{LT}}\right)$, followed by viscous losses within the bronchioles, represented as the small airways flow resistance $\left(FR_{\text{S}}\right)$. Upstream of this region, the airflow passes through the compressible airways, which physiologically correspond to the peripheral airways and the two primary bronchi. Pressure losses within these segments are represented by the compressible airways flow resistance $\left(FR_{\text{c}}\right)$. In the lumped-element representation, this subsystem is modeled using two horizontal cylindrical chambers arranged in parallel. Downstream of the respiratory subsystem, the airflow passes through the trachea and experiences pressure losses quantified by the tracheal flow resistance $\left(FR_{\text{T}}\right)$. The flow then enters the phonatory subsystem, which dynamically modulate the incoming respiratory airflow. This modulation produces a transglottal pressure drop, represented by the vocal fold flow resistance $\left(FR_{\text{VF}}\right)$. The modulated airflow subsequently enters the articulatory subsystem, represented by the upper airways, which extends from the supraglottal region to the oral cavity. Aerodynamic losses that occur within this segment prior to the sound radiation at the lips are represented by the upper airways flow resistance $\left(FR_{\text{U}}\right)$. Finally, the airflow is then expelled through the oral outlet when labial pressure exceeds atmospheric pressure. This effect is modeled by an effective expiratory flow resistance ($FR_{\text{E}}$), which completes the flow resistance network (see Supplementary Materials, Sec. “Lumped-Element System Overview”). Through the serial coupling of these mechanical and aerodynamic elements, the present model provides a complete representation of the energy pathway for phonation.

### Lung Subsystem Parameter Estimation

**Figure 2.**
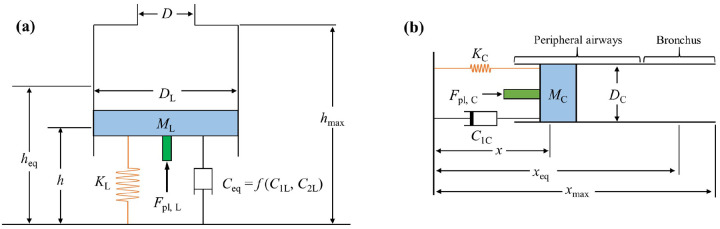
Lumped-element representations of the respiratory subsystem. **(a)** Unified lung model in which the left and right lungs are represented by a single equivalent piston–cylinder system with lumped mass $M_{\text{L}}$. The axial piston position h governs the instantaneous lung volume. Spring stiffness $K_{\text{L}}$ represents elastic recoil of the lung parenchyma, while damping elements ($C_{1\text{L}},C_{2\text{L}}$) capture linear and nonlinear dissipation. The piston is driven by the intrapleural force $F_{\text{pl},\text{L}}$. Geometric parameters $D_{\text{L}},h_{\text{eq}}$, and $h_{\text{max}}$ denote the effective lung diameter, equilibrium position, and maximum piston excursion, respectively. **(b)** Lumped-element model of the compressible airways subsystem representing the peripheral airways and primary bronchi (one assembly shown for clarity). The piston displacement x with mass $M_{\text{C}}$ governs the instantaneous airways volume. Spring stiffness $K_{\text{C}}$ characterizes wall elasticity, while damping $C_{1\text{C}}$ accounts for viscous dissipation. The piston is driven by intrapleural force $F_{\text{pl},\text{C}}$. Geometric parameters $D_{\text{C}},x_{\text{eq}}$, and $x_{\text{max}}$ denote the effective diameter, equilibrium position, and maximum excursion, respectively.

Figure 2a illustrates the lung subsystem modeled with a lumped mass $M_{\text{L}}=1.2\;\text{kg}$^33^. The instantaneous air volume within the lungs ($V_{\text{L}}$) is governed by the piston displacement as follows.

(1) $$V_{\text{L}}=\alpha_{\text{L}}\left(h_{\text{max}}-h\right),$$

where h is the piston position, measured from the reference, and $h_{\text{max}}$ denotes the maximum allowable piston’s traveling distance corresponding to the full lung inflation. The effective cross-sectional area of the piston, $\alpha_{\text{L}}$, was identified as 0.0356 m^2^. The maximum piston displacement was found to be $h_{\text{max}}=19.1\;\text{cm}$ for a total lung capacity of $\text{TLC}=\alpha_{\text{L}}h_{\text{max}}=5.19\;\text{L}$ (see Supplementary materials, Sec. “Lung Geometry and Kinematics”). The elastic behavior of the lung tissue is incorporated into this model through a non-linear spring element and the corresponding elastic restoring force acting on the lung piston is written as

(2) $$F_{\text{el},\text{L}}=-K_{\text{L}}\left(h-h_{\text{eq}}\right),$$

where $K_{\text{L}}$ denotes the effective lung stiffness, which is inversely related to the lung compliance. $h_{\text{eq}}$ represents the equilibrium piston position, which corresponds to the functional residual capacity (FRC) of the lung, defined as the lung volume at which the inward elastic recoil of the lung parenchyma is balanced by the outward recoil of the thoracic cage. The present analysis gives a functional residual capacity of FRC = 2.86 L (see Supplementary materials, Sec. “Lung Spring Model and Compliance”). Along with the elastic force, dissipative forces, representing tissue viscosity, pleural surface friction, and internal structural rearrangements during deformation, also act on the piston and are modeled as

(3) $$F_{\text{damp},\text{L}}=-C_{1\text{L}}\dot{h}-C_{2\;\text{L}}\frac{{\dot{h}}^{3}}{\left|\dot{h}\right|},$$

where $C_{1\text{L}}$ and $C_{2\text{L}}$ denote the linear and nonlinear viscous damping coefficients, respectively. In the present study, particle swarm optimization was used to estimate the lung damping coefficients, resulting in $C_{1\;\text{L}}=195.79\pm 16.05\%\;\text{kg}{\;\text{s}}^{-1}$ and $C_{2\;\text{L}}=1291.01\pm 16.05\%\;\text{kg}{\;\text{m}}^{-2}{\;\text{s}}^{-1}$ (see Supplementary materials, Sec. “Lung Damping Model and Tissue Dissipation” and “Particle Swamp Optimization Algorithm”).

### Compressible Airways Subsystem Parameter Estimation

Figure 2b shows the compressible airways subsystem. Unlike the single piston-cylinder representation of the lungs model, the compressible airways is modeled using two parallel identical piston-cylinder assemblies, with each piston-cylinder unit having a mass of $M_{\text{C}}\;2.95\;\text{g}$ (see Supplementary materials, Sec. “Compressible Airways Geometry and Effective Mass”). The compressible airways volume $V_{\text{C}}$ is related to the piston displacement by

(4) $$V_{\text{C}}\left(t\right)=2\alpha_{\text{C}}\left(x_{\text{max}}-x\left(t\right)\right),$$

where x(t) denotes the instantaneous position of each compressible-airways piston and $x_{\text{max}}$ is the maximum allowable piston excursion corresponding to the fully distended airways configuration. The piston diameter is $D_{\text{C}}=17\;\text{mm}$ and the maximum compressible airways volume $V_{\text{C},\text{max}}$ is 198.65 mL. The elastic behavior of the compressible airways is modeled by a non-linear spring and the corresponding elastic restoring force is expressed as

(5) $$F_{\text{el},\text{C}}=-K_{\text{C}}\left(x-x_{\text{eq}}\right),$$

where $K_{\text{C}}$ denotes the effective compressible airways stiffness and $x_{\text{eq}}=x_{\text{max}}\left(h_{\text{eq}}/h_{\text{max}}\right)$ is the equilibrium piston position (see Supplementary materials, Sec. “ Compressible Airways Spring Model and Compliance”). The dissipative forces, corresponding to the viscous friction within the airways wall and surrounding tissues, act on the compressible airways pistons and is modeled by a Newtonian damper as

(6) $$F_{\text{damp},\text{C}}=-C_{1\text{C}}\dot{x}$$

where $C_{1\text{C}}=7.37{\;\text{Nsm}}^{-1}$ is the compressible-airways damping coefficient (see Supplementary materials, Sec. “Damping Behavior of the Compressible Airways”).

### Vocal Folds Mechanics

**Figure 3.**
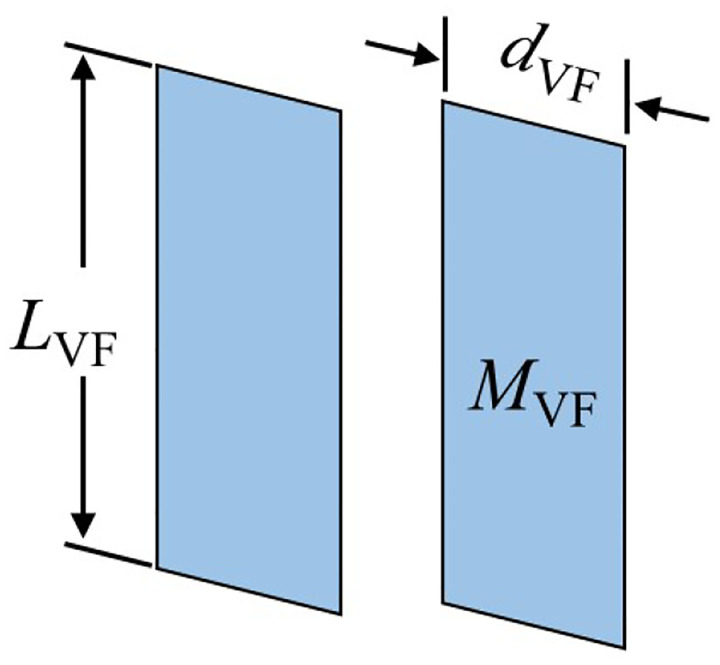
Schematic representation of the single-mass vocal fold model in the present modeling framework. Each vocal fold is represented as a rigid rectangular body with effective mass $M_{\text{VF}}$, superior-inferior length $L_{\text{VF}}$, and medial-lateral thickness $d_{\text{VF}}$. The two folds are positioned symmetrically about the glottal midline.

In the current lumped-element framework, the vocal folds are represented using a single-mass model, as shown in Fig. 3, following the formulation proposed by Ali *et al*.^34^. Accordingly, the superior-inferior length of each vocal fold is taken as $L_{\text{VF}}=1.60\;\text{cm}$, the medial-lateral width is $d_{\text{VF}}=0.32\;\text{cm}$, and the effective mass of each vocal fold is $M_{\text{VF}}=0.120\;\text{g}$. The instantaneous vocal fold volume is defined as $V_{\text{VF}}(t)=A_{g}(t)L_{\text{VF}}$, where $A_{g}(t)$ denotes the time-varying glottal area. For the present modeling work, the glottal area waveform was extracted from laryngeal high-speed videoendoscopy (HSV) recordings of a 49-year-old male subject during sustained phonation of \i\using a machine learning–based segmentation framework (see Supplementary materials, Sec. “Data Collection and Processing”). The elastic response of the vocal folds is modeled using a linear stiffness constant $K_{\text{VF}}=396.6550{\;\text{Nm}}^{-1}$, while viscous dissipation is represented through a nonlinear damping coefficient $C_{\text{VF}}$, expressed as $C_{\text{VF}}=C_{\text{f}}\text{tanh}\;\left(x_{\text{c},\text{VF}}-x_{\text{VF}}\right)$. Here, $C_{\text{f}}=7.5362\times 10^{3}$, and $x_{\text{VF}}$ denotes the instantaneous medial displacement of each vocal fold measured from the midline position, while $x_{\text{c},\text{VF}}$ represents the critical displacement at which the two vocal folds first come into contact. This contact threshold defines the onset of the closed phase. When $x_{\text{VF}}>x_{\text{c},\text{VF}}$, the glottis remains open, when $x_{\text{VF}}=x_{\text{c},\text{VF}}$, the initial contact occurs, and when $x_{\text{VF}}<x_{\text{c},\text{VF}}$, the vocal folds are in collision. Upstream of the glottis, the airflow is driven by the subglottal pressure ($P_{\text{sub}}$), defined as the spatially averaged pressure immediately below the vocal folds. Downstream, the pressure is the supraglottal pressure ($P_{\text{sup}}$), which denotes the pressure immediately above the glottis within the vocal tract. The difference between these pressures constitutes the transglottal driving pressure, which governs the resulting glottal airflow $Q_{\text{g}}(t)$.

### Time-Resolved Phonatory Dynamics Evaluation

**Figure 4.**
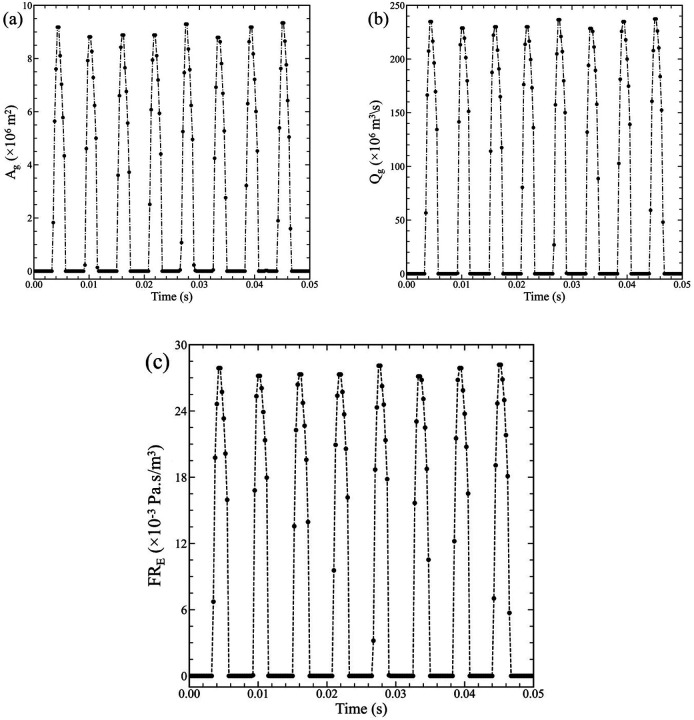
Time-resolved phonatory variables obtained from the unified lumped-element model during the sustained phonation: **(a)** Temporal evolution of the glottal area $\left(A_{g}(t)\right)$ over multiple phonatory cycles, illustrating the periodic opening and closure of the vocal folds; **(b)** Time history of the glottal volumetric flow rate $Q_{g}(t)$ associated with the dynamic modulation of the glottal aperture, reflecting the pulsatile nature of phonatory airflow; and (c) Instantaneous expiratory flow resistance $\left(F_{RE}(t)\right)$ generated by the coupled respiratory-phonatory-articulatory subsystems, capturing the time-varying labial loading.

Having established the unified lumped-element framework, we now examine the time-resolved phonatory variables predicted by the model. to evaluate the model’s ability to reproduce key temporal characteristics of sustained phonation. Figure 4 presents three principal flow-related features: the glottal area waveform, the glottal flow rate, and the time-varying expiratory flow resistance. The temporal evolution of the GAW, shown in Fig. 4a, describes the instantaneous, time-varying area between the left and right vocal folds during phonation over a 50 ms interval. The waveform exhibits a quasi-periodic pattern of repeated glottal opening and closure. The peak glottal area reaches 8–9 mm^2^, while the minimum glottal area reaches zero, indicating a complete glottal closure during each vibratory cycle. The amplitude and periodicity remains stable across the analyzed interval with minor cycle-to-cycle variations in peak amplitude and opening slopes. This indicates small fluctuations in vocal fold biomechanics and the associated aerodynamic loading. The nearly uniform temporal spacing between successive peaks further indicates a stable fundamental frequency.

The glottal flow rate in Fig. 4b reflects the kinematic features of the glottal area waveform. It represents the volumetric airflow passing through the glottis and varies cyclically with vocal fold motion. Each cycle produces a distinct flow pulse that rises rapidly and then decays more gradually. Periods of zero flow occur during complete glottal closure, indicating the absence of leakage with peak flow rates ranging from 200 to 240 ml/s. The waveform exhibits mild asymmetry across different cycles, with small variations in peak magnitude and waveform shape. This signifies subtle fluctuations in the underlying biomechanical and aerodynamic interactions during the sustained phonation.

This glottal flow then passes through the supraglottal vocal tract and is ultimately expelled into the atmosphere. Prior to expulsion, the airflow has to overcome the expiratory flow resistance $\left(FR_{\text{E}}\right)$. Figure 4c shows the temporal evolution of the expiratory flow resistance. Within each phonatory cycle, $FR_{\text{E}}$ rises rapidly from zero to peak values of approximately 25–29 × 10^3^ Pa/(m^3^·s), followed by a more gradual decay back toward zero. This produces a mildly asymmetric pulse shape, characterized by steep rising edges and broader falling segments that coincide with those observed in the glottal flow waveform. Higher glottal flow rates correspond to increased expiratory flow resistance, reflecting the greater pressure required to drive the airflow through the oral outlet. During intervals of zero glottal flow, the expiratory flow resistance collapses to zero, indicating that no pressure gradient is required between the lips and the surrounding atmosphere when the airflow is absent.

**Figure 5.**
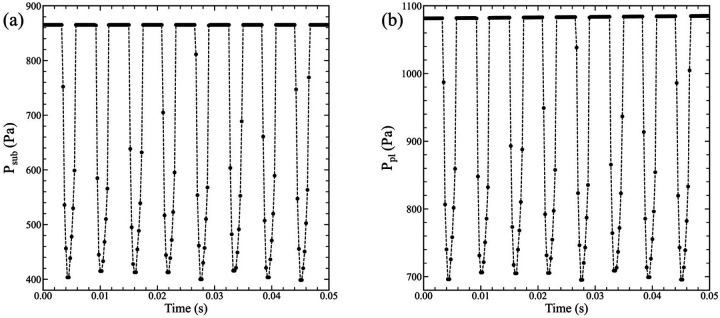
Time-resolved pressure dynamics obtained from the unified lumped-element model during sustained phonation. The figure shows subglottal pressure $P_{\text{sub}}(t)$ within the compressible airways compartment and pleural pressure $P_{\text{pl}}(t)$ applied to the lung piston over multiple oscillation cycles.

The glottal area waveform, glottal flow rate, and expiratory flow resistance waveforms share a common qualitative structure, characterized by a rapid transition from zero to a peak value followed by a decay back to zero within each phonatory cycle. This pattern reflects the opening and closing behavior of the glottal valve and the resulting modulation of airflow and expiratory loading. However, when moving upstream of the vocal folds into the subglottal and respiratory domains, this waveform pattern becomes inverted. Instead of exhibiting isolated pulses that rise from zero, the pressure signals remain elevated for the corresponding time interval of each cycle and undergo sharp decreases during glottal openings. This inverted behavior is clearly observed in the subglottal pressure waveform shown in Fig. 5a. Subglottal pressure serves as the primary aerodynamic driving force for phonation. It is generated by expiratory effort in the lungs and provides the energy required to initiate and sustain the vocal folds vibration. The temporal variation of the subglottal pressure during sustained phonation exhibits a quasi-periodic structure that is synchronized with the glottal flow rate and GAW dynamics. Across the analyzed time window, the subglottal pressure oscillates between 400 Pa and 850–880 Pa. A defining feature of the waveform is the presence of extended pressure plateaus at the upper pressure level, followed by rapid pressure drops and subsequent recovery phases within each phonatory cycle. These high-pressure plateaus occur at uniform intervals. Minor cycle-to-cycle variations are observed in the minimum pressure values and in the slope of the recovery phase. The temporal spacing between successive pressure minima corresponds to the temporal spacing between the maxima of the glottal area waveform. No systematic drift in the mean subglottal pressure level is observed across cycles, suggesting a steady respiratory drive during the recorded phonation.

A similar but more pronounced trend is observed in the intrapleural pressure waveform, shown in Fig. 5b. In comparison to the subglottal pressure, the intrapleural pressure exhibits a higher overall magnitude, reflecting its role as the primary mechanical load responsible for the lung compression and expiratory drive. Over the analyzed time interval, the intrapleural pressure remains within the range of approximately 700 Pa to 1080–1090 Pa. The overall temporal structure of the intrapleural pressure waveform closely parallels that of the subglottal pressure, characterized by extended pressure plateaus followed by rapid decreases and more gradual recovery phases. The elevated plateau levels indicate stronger compressive forces acting on the lungs, which in turn sustain the subglottal pressure necessary for a stable phonation.

## Discussion

The following discussion explains the physical mechanisms underlying the observed temporal patterns of the predicted variables and examines the energy transfer across the coupled respiratory–phonatory–articulatory subsystems during sustained phonation. Each predicted quantity is interpreted in relation to its physiological role and modeling assumptions. Deviations from the experimentally observed fine-scale behavior are also discussed to clarify the implications of these assumptions and to indicate directions for future refinements.

The discussion starts by examining the glottal area waveform, shown in Fig. 4a, as it provides the most direct kinematic description of vocal fold motion. The observed quasi-periodic oscillations arise from nonlinear fluid–structure interaction between the airflow driven by the subglottal pressure and the elastic tissues of the vocal folds, which is consistent with the general vibratory characteristics of the vocal folds^35,36^. Increasing subglottal pressure initiates vocal fold separation, where aerodynamic driving forces dominate over tissue restoring forces. As airflow accelerates through the glottis, intraglottal pressure decreases due to inertial and convective effects^37^. This pressure reduction, together with the elastic recoil of the vocal fold tissues, initiates the closing phase, leading to a complete glottal closure. Apart from minor cycle-to-cycle variations in the peak glottal area and waveform shape, the oscillations remain regular, indicating that the energy supplied by the airflow is balanced by the energy dissipated through tissue damping and collision losses. The absence of irregular oscillations and incomplete closures suggests an adequate vocal fold adduction during sustained phonation.

The glottal flow rate waveform shown in Fig. 4b follows the oscillatory motion of the vocal folds. At the beginning of each phonatory cycle, the vocal folds are fully closed, resulting in maximal flow resistance and zero glottal airflow. During this phase, the expiratory effort leads to the accumulation of pressure beneath the vocal folds. Once the aerodynamic forces overcome the elastic and inertial resistance of the tissues, the glottis begins to open. As the vocal folds separate, the glottal area increases and the resistance to airflow decreases, allowing air to accelerate through the glottal constriction. This produces the rapid rise in glottal flow rate observed at the onset of each cycle. Continued expansion of the glottal opening increases the aerodynamic conductance of the glottis, leading to a further increase in the airflow. Consequently, the peak glottal flow rate occurs at the instant of maximum glottal area, demonstrating the strong influence of glottal geometry on the magnitude and shape of the flow waveform. The relatively consistent peak flow values across cycles indicate stable aerodynamic conditions and effective neuromuscular control of vocal folds’ posture during sustained phonation. Following the peak, the glottal flow rate gradually decreases as the vocal folds enter the closing phase. During this phase, the elastic recoil within the vocal fold tissues, together with reductions in the intraglottal pressure associated with the flow inertia and pressure recovery, initiate and sustain the glottal closure. As the glottal area decreases, the airflow is progressively restricted until the vocal folds come into contact and the flow rate drops to zero. The presence of distinct zero-flow intervals indicates an effective glottal closure, indicating efficient phonatory function.

An important discrepancy is observed between the glottal flow rate predicted by the present model and experimentally reported measurements. Previous studies show that the peak glottal flow does not necessarily coincide exactly with the instant of maximum glottal area^38^. Rather, previous work on glottal inverse filtering and scaled vocal fold models have demonstrated that the peak glottal flow occurrs slightly after the maximum glottal opening^39–41^. This phase lag is attributed to vocal-tract acoustic inertance, which introduces unsteady aerodynamic effects such as a delayed flow buildup and asymmetric flow shutoff. As a result, the airflow does not respond instantaneously to the geometric opening and closing of the vocal folds, leading to a temporal offset between the extrema of glottal area and glottal flow. However, in the present study, glottal flow dynamics are modeled within a lumped-element framework that emphasizes the dominant mechanical and aerodynamic interactions. This formulation therefore neglects distributed acoustic effects and frequency-dependent inertance of the supraglottal vocal tract. Consequently, subtle phase shifts between the glottal area and glottal flow that arise from the acoustic wave propagation, pressure reflections, and inertive loading are not explicitly captured. Despite this simplification, the model successfully reproduces the essential features of glottal flow, including its quasi-periodic structure, effective closure, and stable cycle-to-cycle behaviors. The absence of explicit acoustic inertance therefore highlights future extension of the present work. Incorporating distributed vocal-tract acoustics and inertive effects into the current framework will further refine phase relationships between the glottal area, pressure, and glottal flow.

The time-varying expiratory flow resistance shown in Fig. 4c reflects how the unsteady glottal airflow interacts with the oral outlet during sustained phonation. Unlike resistive elements associated with fixed anatomical constrictions, the expiratory loading arises because the airflow reaching the lips is inherently pulsatile. Even for a nearly constant mouth opening, the instantaneous pressure required to expel air into the surrounding atmosphere varies within each phonatory cycle, as it depends directly on the magnitude and acceleration of the glottal flow arriving at the oral outlet. As seen in Fig. 4c, the expiratory resistance increases during phases of elevated glottal flow, indicating that a greater aerodynamic effort is required to sustain the outflow when the glottal flow rate is higher. As the phonatory cycle progresses and the vocal folds begin to close, the reduction in the glottal flow leads to a corresponding decrease in the expiratory loading. When the glottis is fully closed and the airflow ceases, the expiratory resistance effectively vanishes, indicating that no pressure difference is required to maintain the outflow in the absence of flow. In the present framework, the expiratory resistance does not act as an independent control element but instead emerges as a downstream response to the pulsatile flow generated at the glottis. Small variations in the magnitude and timing of the resistance waveform primarily reflect variations in the upstream glottal flow, reinforcing the interpretation that expiratory loading is governed by instantaneous flow conditions. However, it should be noted that, in real life phonation, the pressure response at the lips is also influenced by distributed acoustic propagation and inertive effects within the vocal tract, which can introduce phase delays between changes in the glottal flow and supraglottal pressure. These effects are not explicitly resolved in the present lumped-element model. Nevertheless, the behavior shown in Fig. 4c captures the dominant, the cycle-resolved relationship between the glottal flow and the expiratory flow resistance.

The subglottal pressure waveform shown in Fig. 5a reflects the dynamic balance between respiratory driving forces and the time-varying aerodynamic load imposed by the vocal folds vibration. The subglottal pressure acts as the primary energy source for phonation, and during the sustained phonation, it is modulated by the cyclic opening and closing of the glottis. The extended plateaus observed at elevated pressure levels correspond to phases of complete glottal closure. During these intervals, the airflow is strongly impeded, and the continued expiratory effort leads to the accumulation of pressure within the subglottal system. These plateau regions therefore represent periods of energy storage, during which the elastic recoil of the lungs sustains a nearly constant driving pressure. In physiological phonation, however, such plateaus are not perfectly flat, as subglottal pressure typically exhibits small oscillations even during the apparent closure. The absence of these fine-scale pressure fluctuations in the present results arises from the modeling assumptions adopted in this work. In the present work, the vocal folds are represented using a single-mass lumped-element model in which a complete vocal fold contact is assumed whenever the glottal area measured at the superior edges reaches zero. This assumption provides a clear and computationally efficient representation of glottal closure. However, this approach fails to capture the three-dimensional nature of vocal folds’ motion. In realistic phonation, glottal opening and closing do not occur simultaneously along the entire inferior–superior extent of the vocal folds. Instead, separation and contact progress gradually, initiating at the inferior edges and propagating toward the superior edges. Our HSV recordings provide measurements of the glottal area only from a superior viewing perspective. As a result, a zero glottal area in the extracted glottal area waveform corresponds specifically to contact at the superior edges of the vocal folds. When this occurs, the present model assumes that the entire medial surfaces of the vocal folds are in contact. In reality, during the opening phase, the vocal folds start to separate from each other at the inferior edges while the superior edges remains closed. Therefore, even when the glottal area measured at the superior edge is zero, volumetric changes is already happening in the inferior portion of the glottis. These subglottal-to-infraglottal geometric changes allow the airflow acceleration and pressure redistribution below the vocal folds, leading to subtle oscillations in the subglottal pressure. Since the current modeling framework relies on two-dimensional glottal area measurements extracted from the superior edges only, it lacks direct information about the dynamic behavior of the glottal geometry at the inferior edge. As a result, the model is inherently blind to volumetric changes and pressure fluctuations occurring behind the closed superior edges. As a consequence of this limitation, the model predicts an exactly constant subglottal pressure during the closed phase, while in real life phonation, the subglottal pressure displays cycle-synchronous oscillations. Although the current framework does not capture every temporal nuance associated with the three-dimensional vocal fold motion, the time-dependent behavior obtained in the model represents a physiologically meaningful approximation of the underlying respiratory mechanics. From an applicational point of view, the resulting subglottal pressure waveform can be used as an inlet condition for aerodynamic and aeroacoustic modeling of phonation. In most of the existing literature, subglottal pressure is assumed to be constant for such modeling work^42–46^. This assumption can hide key aspects of flow–structure interaction, energy exchange between the airflow and the vocal folds, and parameters associated with voice quality. By capturing the time-varying behavior of the subglottal pressure, the present framework serves as a strong foundation for subject-specific phonation modeling and for future extensions of the work that will incorporate more detailed three-dimensional and acoustic effects.

The intrapleural pressure waveform shown in Fig. 5b characterizes the loading conditions imposed on the lungs during sustained phonation. Within the respiratory system, variations in intrapleural pressure directly regulate the lung volume by controlling the compressive forces acting on lung tissues. A change in the intrapleural pressure therefore corresponds to a change in the compressive load on the lungs. In our present lumped-element modeling, the intrapleural pressure is the primary driving force, which links the respiratory mechanics with the glottal airflow. Thus, the temporal variation of intrapleural pressure arises from the strong coupling between the respiratory subsystem and the oscillatory dynamics of the vocal folds. As the vocal folds periodically restrict and release airflow at the glottis, the downstream aerodynamic load experienced by the respiratory system varies, producing corresponding modulations in intrapleural pressure. During phases of increased glottal opening, the overall airways resistance decreases, allowing the airflow to pass more readily through the system. As a result, a lower driving pressure is required at the level of the lungs, leading to a reduction in intrapleural pressure. Conversely, as the glottis approaches closure and the airways resistance increases, a higher intrapleural pressure is necessary to sustain the expiratory airflow. This cycle-dependent adjustment reflects the dynamic redistribution of pressure and energy across the coupled respiratory-phonatory system during sustained phonation. Similar to the subglottal pressure, extended upper plateaus are observed in the intrapleural pressure waveform, which would not be expected in real-life phonation. As in the case of the subglottal pressure, this behavior arises from the limitations of the present one-dimensional modeling framework, which does not resolve fine-scale pressure fluctuations. Thus, these plateau regions are interpreted as intervals during which the respiratory system maintains a quasi-steady operating state. One prominent approach to further extend the proposed modeling paradigm is to take into account the power-law rheology and viscoelasticity of bio-tissues. This will be looked into in our future works, following our earlier developmental research in applied and computational mathematics^47–51^.

## Methods

### Data Collection and Processing

A fundamental requirement of our computational framework is that the motion of the spring-mass-damper system representing the vocal folds should capture their sustained vibration. In particular, the timing and magnitude of the oscillations in the model must correspond to the opening and closing phases of the glottis. To capture this dynamic behavior, we employed the experimental data of glottal area waveform to capture temporal variations in the glottis. For this purpose, we analyzed laryngeal high speed videoendoscopy (HSV) data from a 49-year-old male participant with a normophonic voice. The data collection was conducted under an approved IRB at Mayo Clinic-Arizona in accordance with human research guidelines, and informed consent was obtained from the participant. The data analysis of the de-identified data was done under an exempt IRB at Michigan State University. Recordings were obtained transnasally using a flexible nasolaryngoscope coupled to a high-speed camera operating at 4000 frames per second. The image sequences were preprocessed to enhance contrast and reduce noise and a U Net-based convolutional neural network was then used to segment the glottal area frame by frame. The resulting glottal area waveform $A_{g}(t)$ provides the time varying glottal aperture that serves as input to the lumped-element phonatory model and underlies all subsequent simulations of flow and pressure. See Supplementary materials, section “Data Collection and Processing”, for the detailed descriptions of participant recruitment, imaging parameters, preprocessing steps, network architecture training, procedure and quality control measures.

### Numerical Method

The dynamics of the coupled respiratory–phonatory-articulatory subsystems are governed by the nonlinear force balance equation as given in equation (7) (see Supplementary materials, section “Numerical Method” for the detailed derivation).


(7) $$\frac{M_{L}}{\alpha_{L}^{2}}{\ddot{V}}_{L}(t)-\frac{M_{C}}{2\alpha_{C}^{2}}{\ddot{V}}_{C}(t)+FR_{S}{\dot{V}}_{L}(t)+\frac{C_{1L}}{\alpha_{L}^{2}}{\dot{V}}_{L}(t)-\frac{C_{1C}}{2\alpha_{C}^{2}}{\dot{V}}_{C}(t)+\frac{C_{2L}}{\alpha_{L}^{3}}\frac{{\dot{V}}_{L}(t{)}^{3}}{\left|{\dot{V}}_{L}(t)\right|}+\frac{K_{L}(t)}{\alpha_{L}}\left(\frac{V_{L}(t)}{\alpha_{L}}+h_{\text{eq}}-h_{max}\right)-\frac{K_{C}(t)}{\alpha_{C}}\left(\frac{V_{C}(t)}{2\alpha_{C}}+x_{\text{eq}}-x_{max}\right)-\frac{M_{L}g}{\alpha_{L}}+\frac{R_{L}}{\alpha_{L}}-\frac{R_{C}}{\alpha_{C}}=0$$

The lung and compressible airways volumes are kinematically coupled through the effective glottal airflow rate $\left({\tilde{Q}}_{g}(t)\right)$ via the following volumetric continuity constraint

(8) $${\dot{V}}_{L}\left(t\right)+{\dot{V}}_{C}\left(t\right)=-{\tilde{Q}}_{g}\left(t\right),$$

which implies

(9) $${\dot{V}}_{C}\left(t\right)=-{\tilde{Q}}_{g}\left(t\right)-{\dot{V}}_{L}\left(t\right),\;{\ddot{V}}_{C}\left(t\right)=-{\dot{\tilde{Q}}}_{g}\left(t\right)-{\ddot{V}}_{L}\left(t\right),$$

Substituting equation (9) into equation (7) eliminates the volumetric terms corresponding to the compressible airways and gives a reduced-order equation of motion, written in the following general form

(10) $${\ddot{V}}_{L}(t)=ℱ\left(t,V_{L}(t),{\dot{V}}_{L}(t),{\tilde{Q}}_{g}(t),{\dot{\tilde{Q}}}_{g}(t)\right)$$

where ℱ(⋅) represents the nonlinear contribution of lung elasticity, damping, inertia, gravity, airways resistance, and flow-induced forcing. To numerically integrate equation (10), the second-order system is recast as a first-order system by defining the state variables

(11) $$y_{1}(t)=V_{L}(t),\;y_{2}(t)={\dot{V}}_{L}(t),$$

leading to

(12) $$\frac{d}{dt}\left[\begin{array}{l} y_{1}\\ y_{2} \end{array}\right]=\left[\begin{array}{c} y_{2}\\ ℱ\left(t,y_{1},y_{2},{\tilde{Q}}_{g}(t),{\dot{\tilde{Q}}}_{g}(t)\right) \end{array}\right].$$

The system is integrated in time using a fourth-order Runge-Kutta (RK4) scheme. Given the state $y_{n}$ at time $t_{n}$, the update to $t_{n+1}=t_{n}+\Delta t$ is obtained as

(13) $$y_{n+1}=y_{n}+\frac{\Delta t}{6}\left(k_{1}+2k_{2}+2k_{3}+k_{4}\right),$$

where the stage vectors $k_{i}$ are computed in the standard RK4 manner using the right-hand side of equation (12). The effective glottal flow rate ${\tilde{Q}}_{g}(t)$ and its time derivative ${\dot{\tilde{Q}}}_{g}(t)$ are evaluated at intermediate Runge-Kutta stages using linear interpolation to ensure consistency with the continuous-time formulation. The compressible airways volume rate ${\dot{V}}_{C}(t)$ is reconstructed exactly at every time step from equation (8), ensuring the discrete conservation of volumetric flow. The time step is fixed at $\Delta t=2.5\times 10^{-4}\;\text{s}$, corresponding to the temporal resolution of the high-speed imaging data (4000 frames per second). The initial conditions are prescribed as $V_{L}(0)=3.0\times 10^{-3}{\;\text{m}}^{3}$ and ${\dot{V}}_{L}(0)=0{\;\text{m}}^{3}/\text{s}$, while the initial compressible airways volume satisfies $V_{C}(0)=V_{C,\text{max}}$ and ${\dot{V}}_{C}(0)=-{\tilde{Q}}_{g}(0)-{\dot{V}}_{L}(0)$, following the volumetric continuity constraint at t=0.

### Validation

The predictive performance of the unified lumped–element model was assessed by comparing its subglottal pressure predictions with excised-larynx measurements obtained by Lehoux *et al*.^16^. Their experiment used a red deer larynx mounted on a straight Plexiglas tube configured as an anechoic, purely resistive subglottal tract. The red deer larynx is widely regarded as an excellent biomechanical surrogate for the human larynx because its geometry, tissue composition, and vibratory behavior closely mimic those of adult male human vocal folds, making it a standard model for phonatory mechanics.

During steady phonation in the reference experiment, the subglottal pressure was approximately 2500Pa, with oscillation frequencies between 100–120Hz. To replicate these conditions, the present model was driven with an effective pressure input $\delta_{P}=2700\text{Pa}$, producing a simulated subglottal pressure waveform immediately below the glottis. The vocal-fold kinematics were prescribed from the HSV-derived glottal area waveform, yielding an oscillation frequency of 127.2Hz. For comparison, both model-predicted and experimental waveforms were nondimensionalized over a single phonatory cycle.

The agreement was evaluated using the point-wise relative error $e(t)=\left|P_{\text{num}}(t)-P_{\text{exp}}(t)\right|/P_{\text{exp}}(t)\times 100$. The maximum discrepancy ($e_{\text{max}}=13.33\%$) occurs near peak glottal opening, where the experimental flow exhibits a three-dimensional jet separation and vortical structures that cannot be captured by a one-dimensional lumped-element representation. Despite this localized deviation, the model reproduces the canonical pressure waveform pattern, including the closed-phase plateau, the monotonic pressure drop during the opening, and the subsequent rise during the closing. The timing and amplitude of the oscillations align closely with measurements, and the mean cycle-averaged error is only $e_{\text{mean}}=2.96\%$, demonstrating that the model reliably captures the dominant subglottal pressure dynamics.

## Supplementary Material

Supplementary Files

This is a list of supplementary files associated with this preprint. Click to download.

- NatureScientificReportsSupplementaryMaterial.pdf

## Figures and Tables

**Figure 6.**
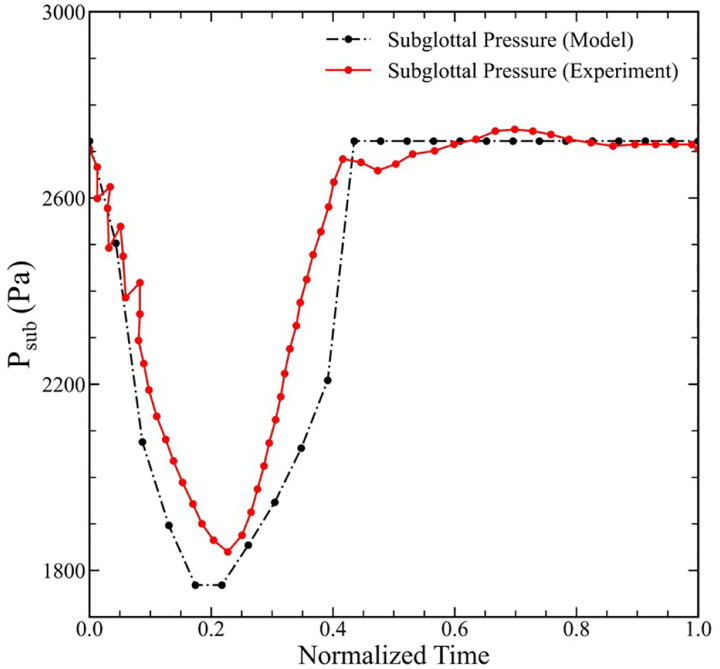
Comparison of the subglottal pressure waveform predicted by the unified lumped-element model with experimentally measured subglottal pressure from excised-larynx experiments reported by Lehoux *et al*.^16^. Both waveforms are shown over a normalized phonatory cycle.

## Data Availability

The data supporting the findings of this study are available from the corresponding author upon reasonable request. The HSV recordings contain potentially identifiable information and are not publicly available due to privacy restrictions.
